# Aspirin is Involved in the Cell Cycle Arrest, Apoptosis, Cell Migration, and Invasion of Oral Squamous Cell Carcinoma

**DOI:** 10.3390/ijms19072029

**Published:** 2018-07-12

**Authors:** Xiaoqi Zhang, Hao Feng, Ziyu Li, Jie Guo, Minqi Li

**Affiliations:** 1 Shandong Provincial Key Laboratory of Oral Tissue Regeneration, Department of Bone Metabolism, School of Stomatology Shandong University, Jinan 250012, China; zhangxiaoqi67@163.com (X.Z.); kqyxyfh@163.com (H.F.); liziyu9630@163.com (Z.L.); 2 Shandong Provincial Key Laboratory of Oral Tissue Regeneration, Department of Orthodontics, School of Stomatology Shandong University, Jinan 250012, China; kqgj@sdu.edu.cn

**Keywords:** oral squamous cell carcinoma, cell cycle, invasion, migration, apoptosis, bioinformatics

## Abstract

Oral squamous cell carcinoma (OSCC) is one of the most common cancers worldwide. In China, its 5-year survival rate is roughly 50%, owing to acquired chemotherapeutic resistance and metastasis of the disease. Accumulating evidence demonstrates that aspirin (ASA) acts as a preventive or therapeutic agent in multiple cancers; however, anti-tumor activities induced by aspirin are unclear in OSCC. To investigate the possible role of aspirin in OSCC development, we first employed bioinformatics to analyze the anti-OSCC effects of aspirin. We performed a genetic oncology (GO) enrichment analysis using the Database for Annotation, Visualization, and Integrated Discovery (DAVID), and the protein–protein interaction (PPI) network analysis by Cytoscape for differentially expressed genes (DEGs). We also evaluated the potential effects of aspirin on cell proliferation, invasion, migration, and apoptosis in two well-characterized OSCC cell lines (TCA8113 and CAL27). The bioinformatic results revealed that aspirin could inhibit proliferation by blocking the cell cycle, and could reduce migration and invasion via the PI3K-Akt and focal adhesion pathways. We found that ASA could downregulate the OSCC cell proliferation colony formation, invasion, and migration, as well as upregulate apoptosis. Furthermore, we found that ASA suppressed the activation of the focal adhesion kinase (FAK) and the phosphorylation of Akt, NF-κB, and STAT3. Overall, our data suggested that ASA may be developed as a chemopreventive agent to effectively treat OSCC.

## 1. Introduction

Oral squamous cell carcinoma (OSCC) is currently the sixth most common malignancy worldwide and ranks eighth in cancer-related mortalities [1]. OSCC has a well-characterized progression, from hyperplastic proliferation through to dysplasia and carcinoma formation. Its progression involves complex processes including the accumulation of numerous genetic and epigenetic oncogenes and suppressor genes, resulting in the dysregulation of multiple signaling pathways that disrupt the cell cycle as well as the balance between the cell proliferation and cell death [2]. Although surgical therapy, in combination with necessary radiation and/or chemotherapy, are widely used in OSCC patients, OSCC is characterized by a poor prognosis and reduced survival rates. In the past decades, the five-year survival rate of the OSCC patients still remains significantly low; it has remained at 50–60%, despite surgical, chemotherapeutic, and radiotherapeutic advancements [3]. Clinical and laboratory studies suggested that OSCC cells have become resistant to clinical chemotherapeutic agents [4]. However, it usually takes many years for malignant changes to develop in epithelial cells, in which they undergo multiple cellular and genetic alterations, making OSCC an optimal disease for pharmacological interventions, before the cancer transformation. Chemoprevention has been considered a rational and appealing strategy to prevent or delay the development of OSCC [5].

Experimental tumor model studies show that non-steroidal anti-inflammatory drugs (NSAIDs) impair the growth and development of head and neck (HN) SCC, indicating it potential use as a chemopreventive agent [6,7]. Aspirin (ASA), an NSAID, is a well-known antipyretic and analgesic agent and is used to prevent recurrent transient ischemic attacks or strokes [8]. In addition to its classical anti-inflammatory function, clinical and epidemiological studies indicate that aspirin can be used as a preventive or therapeutic agent in multiple cancers, including oral cancer [9,10,11,12]. While the exact mechanism through which NSAIDs contribute to chemoprevention is not completely understood, it is thought to involve the inhibition of the enzyme cyclooxygenase-2(COX-2) of the arachadonic acid metabolism pathway [13]. Studies have demonstrated that the levels of COX-2 and its prostaglandin derivatives, specifically prostaglandin E2 (PGE2), are increased in premalignant and malignant lesions of HNSCC [14]. The overexpression of COX-2 and hence the increased levels of PGE2 in HNSCC patients could contribute to carcinogenesis via multiple mechanisms such as inhibiting apoptosis and promoting invasion and metastasis. However, facing mounting evidence, the contribution of the COX-independent pathways to the anticancer effects of aspirin (or its metabolite, salicylate) cannot be discounted. Cancer cells manage to defend themselves from radiation or anticancer agents by the activation of NF-κB [15]. Therefore, the suppression of NF-κB activity in cancer cells may be crucial to induce a marked cell death by chemotherapeutic agents. Thus, ASA has been found to inhibit IκB kinase β and prevent NF-κB activation both in vivo and in vitro [16,17].

In recent years, microarrays, based on high throughput platforms, unveiled themselves as promising and efficient tools when searching for meaningful genes and epigenetic alternations in carcinogenesis, as well as being helpful to identify the biomarkers used for diagnosis or prognosis [18]. Gene expression profile microarrays have been widely used when locating differentially expressed genes (DEGs) in OSCC [19]. However, single studies have limited numbers of overlapping gene profiles and have been insufficient when identifying the key genes and pathways concerned with multiple biological processes and molecular functions. By using integrated and advanced bioinformatical analysis for the available microarray data, it may reveal more reliable and precise results by overlapping the relevant datasets. In this study, we overlapped two datasets to uncover more underling information and put forward a better understanding of the OSCC treated by aspirin.

This study investigates the role of ASA in the pathobiology of OSCC and shows that ASA treatment can effectively promote apoptosis and reduce the proliferation, migration, and invasion of OSCC cells. We believe this study enhances our knowledge of the pathogenesis of this malignancy, and potentially helps to elucidate the underlying mechanism of ASA involved in OSCC.

## 2. Results

### 2.1. Identification of the DEGs

**Figure 1:**
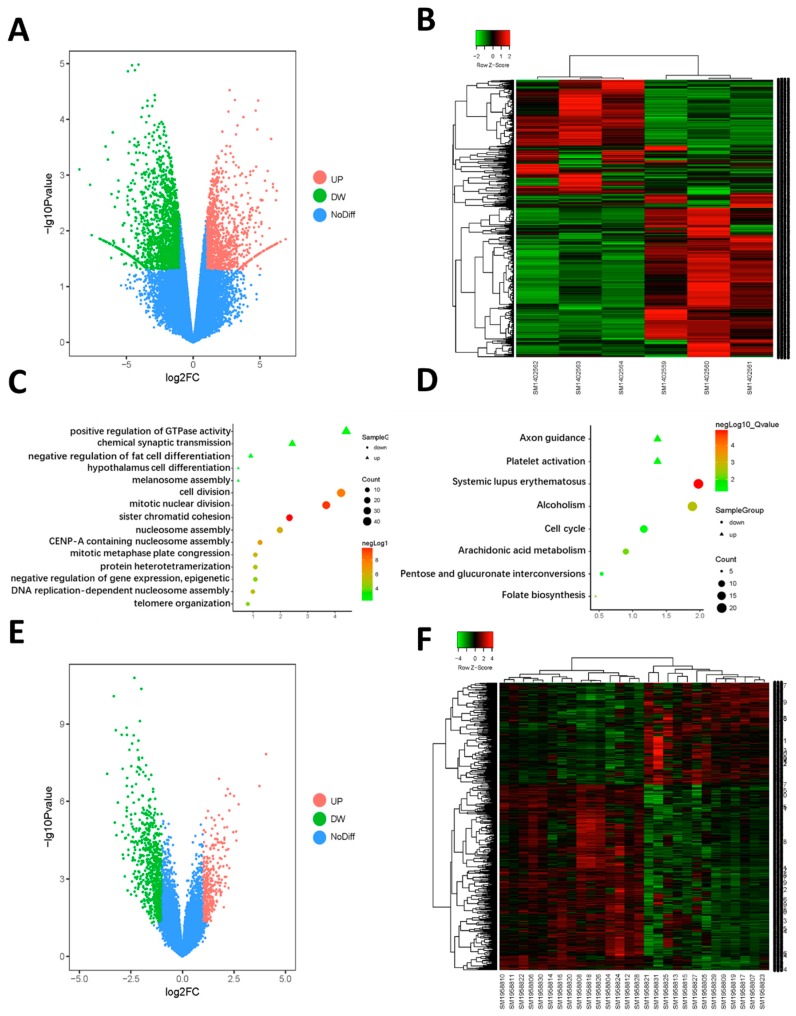
(**A**) Volcano plot visualizing differentially expressed genes (DEGs) in GSE58162 (three groups of control samples and three groups of aspirin treated samples). The vertical lines demark the fold change values. The right vertical line corresponds to ≥2-fold up and the left vertical line ≥2-fold down changes, while the horizontal line marks a −log10p-value of 0.05. (**B**) Heat map hierarchical clustering reveals DEGs in aspirin treated groups compared with control groups. (**C**) Functional enrichment analysis of DEGs in GSE58162. Significantly enriched biological processes were ranked by *p*-value by using the Database for Annotation, Visualization, and Integrated Discovery (DAVID). A *p*-value <0.05 was regarded significant. (**D**) KEGG pathway enrichment analysis of DEGs in GSE58162. Significantly enriched pathways were ranked by *p*-value, using DAVID. A *p*-value <0.05 was regarded significant. (**E**) Volcano plot visualizing DEGs in GSE75538 (14 oral squamous cell carcinoma (OSCC) samples and 14 matched normal samples). (**F**) Heat map hierarchical clustering reveals DEGs in OSCC groups compared with normal groups.

**Figure 2:**
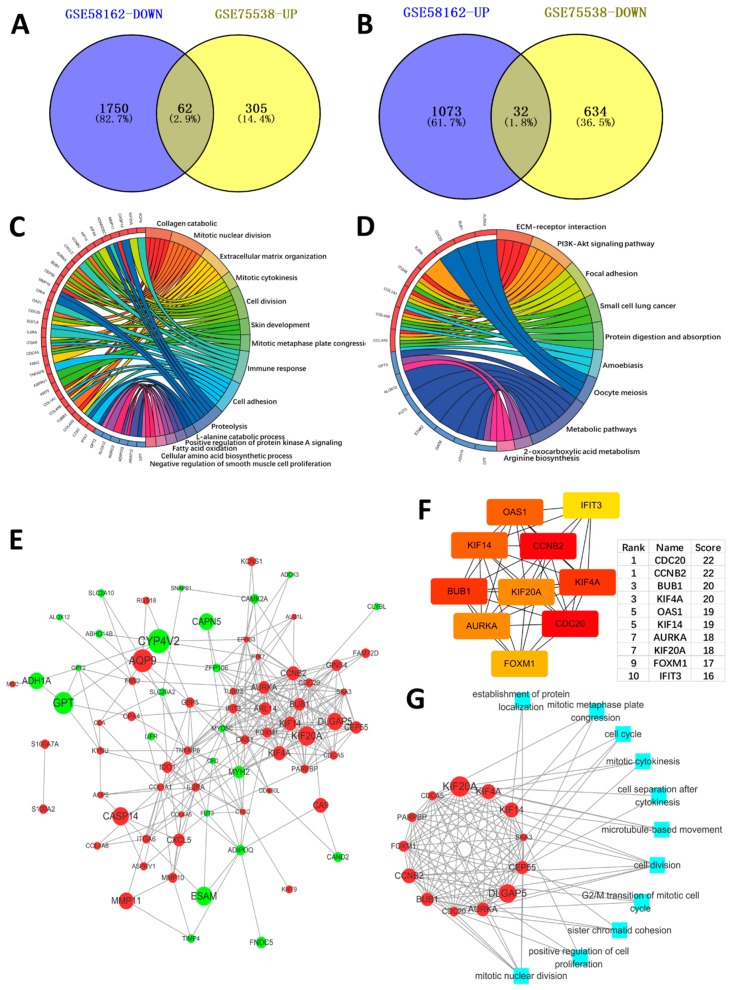
(**A**) The down regulated genes in GSE58162 (genes were down regulated after aspirin treated in OSCC cell line) and the up regulated genes in GSE75538 (genes were high expressed in tumor samples compared with normal samples) were overlapped by the Venn diagram. There outcome was 62 genes, which were highly expressed in OSCC and could be down regulated by aspirin. (**B**) The up regulated genes in GSE58162 and the down regulated genes in GSE75538 were overlapped by the Venn diagram. The outcome showed that there were also 32 genes, which were low expressed in OSCC and could be up regulated by aspirin. (**C**) GO enrichment analysis of the overlapped 62 and 32 genes (biologic process). (**D**) KEGG pathway analysis of the overlapped 62 and 32 genes. (**E**) The protein–protein interaction (PPI) network of the overlapped genes. The red represented the high expressed genes in OSCC, which were down regulated by aspirin, and the green represented the low expressed genes in OSCC, which were up regulated by aspirin. The node size represented |logFC|. (**F**) The top 10 hub genes in the network, which were ranked by node degree. (**G**) The core module of the network and its function.

GEO2R analyzed every dataset microarray respectively and screened the DEGs. By using *p* <0.05 and logFC >|1| as the cut-off criterion, there were 1105 genes up regulated and 1812 genes down regulated in GSE58162 as DEGs (Figure 1A,B). Meanwhile, there were 367 genes up-regulated and 666 genes down regulated in GSE58162 (Figure 1E,F). We obtained 62 genes that were high-expressed in OSCC, but could be down regulated by aspirin by using Venn diagrams to overlap the down-regulated DEGs in GSE58162 and the up-regulated DEGs in GSE75538 (Figure 2A). Furthermore, we obtained 32 genes that were low-expressed in OSCC, but could be up regulated by aspirin by using Venn diagrams to overlap up-regulated DEGs in GSE58162 and down regulated genes in GSE75538 (Figure 2B). 

### 2.2. Function Enrichment Analysis

We performed an enrichment analysis to explore the biological process and pathway that aspirin affected. The genetic oncology (GO) enrichment of the biological process (BP) was conducted by the Database for Annotation, Visualization, and Integrated Discovery (DAVID). Figure 1C showed biological changes after being treated with aspirin in the SCC25 cell line. We found that the down regulated genes were significantly enriched in the regulation of mitosis and cell cycle. This indicated that aspirin could affect the proliferation of SCC25 cell lines. As for the pathways (Figure 1D), we also found that the cell cycle pathway was down regulated, which meant that aspirin could block the cell cycle and decrease the SCC25 cell proliferation.

Furthermore, we found that the 62 high-expressed genes in OSCC, which were down-regulated by aspirin, were enriched in the regulation of cell proliferation, cell adhesion and ECM catabolic (Figure 2C). These biological processes were concerned with OSCC malignant phenotypes, such as cancer cell proliferation, migration, invasion, and metastasis. The genes involved in these biological processes were down-regulated by aspirin, which meant that aspirin has an anti-tumor effect. Aspirin can inhibit these malignant biological processes of OSCC. The results of the KEGG analysis (Figure 2D) showed that the extracellular matrix (ECM)-receptor interaction, PI3K-Akt pathway, and the focal adhesion pathway were mainly involved, indicating that aspirin has effects in these pathways. 

### 2.3. PPI Network Construction, Significant Modules Identification, and Modules Analysiss

We used the online database STRING to construct the PPI network. Figure 2E was the PPI of the 62 highly-expressed genes in OSCC, which were down-regulated by aspirin, and the 32 low-expressed genes in OSCC, which were up-regulated by aspirin genes. We used Cytohubba in Cytoscape to uncover the hub genes in the network. The results are showed in Figure 2F. The hub genes were high degree (node connection, a node connects with other nodes, its degree was higher), which means these genes have a key role in the network. We found that almost all of the hub genes were concerned with cell proliferation. Figure 2G showed the core module of the network and its function. Most of its functions were involved in cell proliferation. All of these results indicated the aspirin has an anti-proliferation effect in OSCC. For the 10 hub genes, a GSEA analysis was performed to revealed their function (Figure S2).

### 2.4. Aspirin Inhibits Cell Viability in a Dose- and Time-Dependent Manner

**Figure 3:**
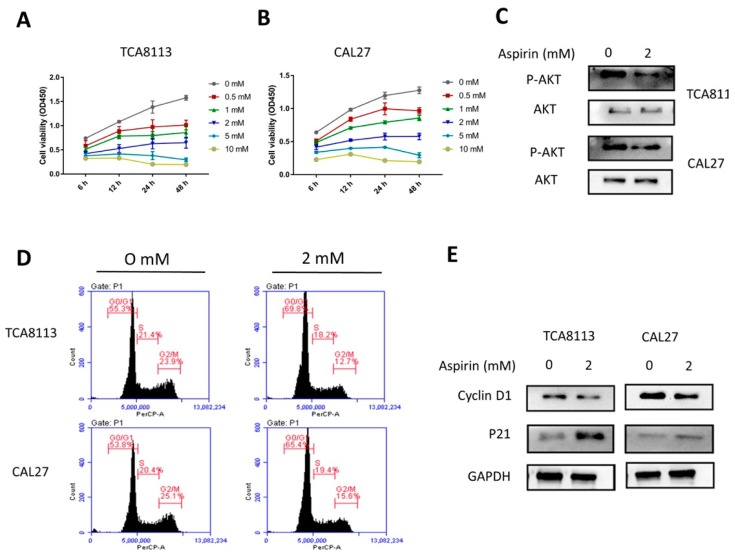

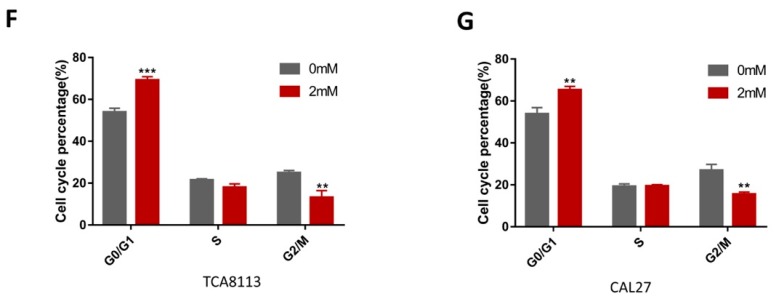
Aspirin inhibited cell viability and promoted cell cycle G0/G1 phase arrest. (**A**,**B**) Aspirin (ASA) inhibited cell viability in dose- and time-dependent manner. The TCA8113 and CAL27 cells were treated with different concentrations of aspirin for the indicated times, and the viability of these cells was measured using CCK8 assays. (**C**) The ASA treatment affected the phosphorylation of Akt in TCA8113 and CAL27 cells. Western blotting was used to detect the expression and phosphorylation of Akt with ASA treatment. (**D**–**G**) The cell cycle was arrested in the G1 phase with ASA treatment the in TCA8113 and CAL27 cells. Flow cytometry was used to analyze the effects of ASA on the cell cycle when cells were treated with or without ASA for 24 h. (**E**) ASA promoted the expression of p21 and decreased the expression of cyclin D1 in the TCA8113 and CAL27 cells. After the cells were incubated with 2 mM ASA for 24 h, the protein levels were detected by Western blotting using indicated antibodies, and GAPDH was used as a loading control. ** *p* < 0.01 and *** *p* < 0.005. The data were presented as the mean ± standard deviation (SD) (*n* = 3).

To determine the effect of ASA on cell viability in the OSCC cell lines, TCA8113 and CAL27 cells were treated with different concentrations of ASA for the indicated times. As shown in Figure 3A,B, after two cell lines were treated with ASA (0–10 mM) for 6, 12, 24, and 48 h, the cell viability was inhibited in a dose- and time-dependent manner, compared to that of the untreated group. Since the ASA treatment at 1, 2, 5, and 10 mM showed strong inhibitory effects on the proliferation of TCA8113 and CAL27 cells, 2 mM was chosen as the representative dose for the in vitro treatment in subsequent studies. To understand the mechanism of the inhibition of ASA, we investigated the effect of ASA on Akt signaling. The results of both the TCA8113 and CAL27 cells showed that the phosphorylation of Akt was significantly suppressed after the treatment of ASA (Figure 3C, Figure S1C).

### 2.5. Aspirin-Induced G0/G1 Arrest

The proliferation inhibition of ASA could be due to the cell-cycle arrest; therefore, the cell cycle analysis was conducted using flow cytometry. After being treated with ASA, the cell cycle distribution analysis showed significantly increased cell populations in the G0/G1 phase and decreased cell populations in the G2/M phase of TCA8113 (Figure 3D,F). Similar effects were observed in CAL27 (Figure 3D,G). These results suggested that the growth inhibition with the ASA treatment might be associated with its ability to induce cell growth-arrest in the G0/G1 phase. To further understand the mechanism of the cell cycle arrest, the expression levels of the cell cycle regulatory proteins were analyzed by the Western blot analysis. As shown in Figure 3E (Figure S1A,B), the ASA treatment specifically decreased the expression of cyclin D1 and enhanced the expression of p21.

### 2.6. Aspirin Induces Apoptosis in TCA8113 and CAL27 Cells

**Figure 4:**
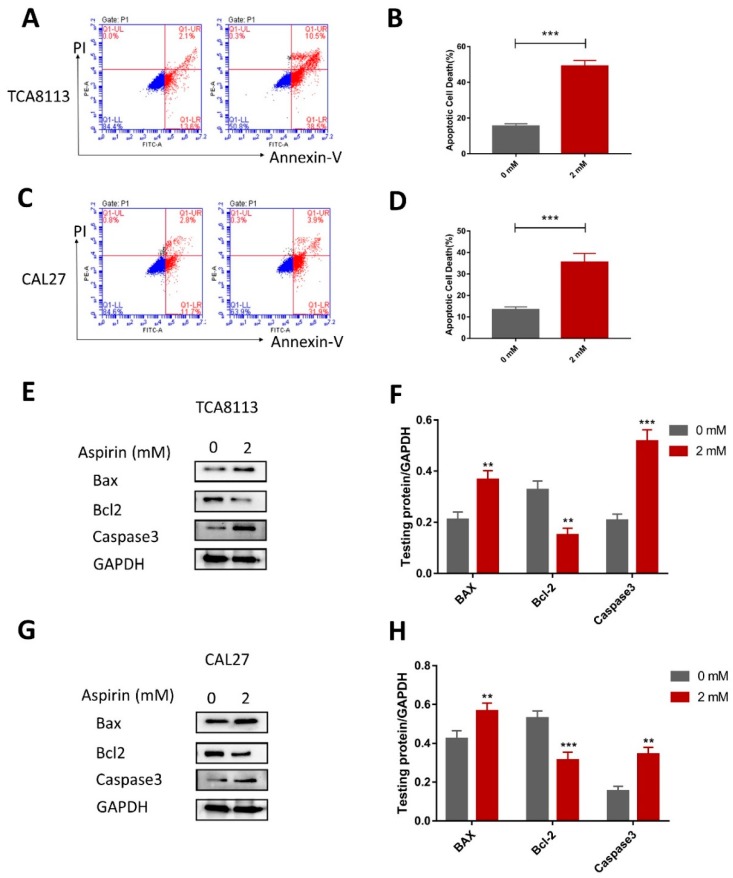
Aspirin promoted apoptosis in the TCA8113 and CAL27 cells. (**A**,**B**) The apoptosis cell rates were increased with ASA treatment in the TCA8113 cells. The apoptotic cells were detected by flow cytometry after staining with Annexin V and PI after cells were treated with 2 mM ASA for 24 h. (**C**,**D**) Similar results were observed in CAL27. (**E**,**F**) The ASA treatment increased the expression of Bax and caspase3, and inhibited the expression of Bcl-2. Apoptosis related protein levels were detected by Western blotting using indicated antibodies, and GAPDH was used as a loading control. (**G**,**H**) Similar results were observed in CAL27. ** *p* < 0.01 and *** *p* < 0.005. The data were presented as the mean ± SD (*n* = 3).

As we observed a significant inhibitory effect of ASA on squamous carcinoma TCA8113 and CAL27 cells, we investigated whether the ASA could induce apoptosis in OSCC cells by Annexin V and PI double staining. The effect of ASA on the apoptosis of the TCA8113 and CAL27 cells, as detected by flow cytometry; the ASA treatments for 24 h resulted in over 49% of apoptotic cells in the TCA8113. Furthermore, the baseline apoptosis of the solvent control cells was almost 15% (Figure 4A,B). Similar effects were observed in the CAL27 cells (Figure 4C,D). These results indicated that ASA could induce apoptosis in the TCA8113 and CAL27 cells. Moreover, as shown in Figure 4E,F, ASA could enhance the expression of Bax and caspase3, as well as inhibit the expression of Bcl-2, which indicated that ASA triggered a caspase cascade. Similar effects were observed in CAL27 (Figure 4G,H).

### 2.7. Aspirin Inhibits Migration, Invasion and Cloning Formation in TCA8113 and CAL27 Cells

**Figure 5:**
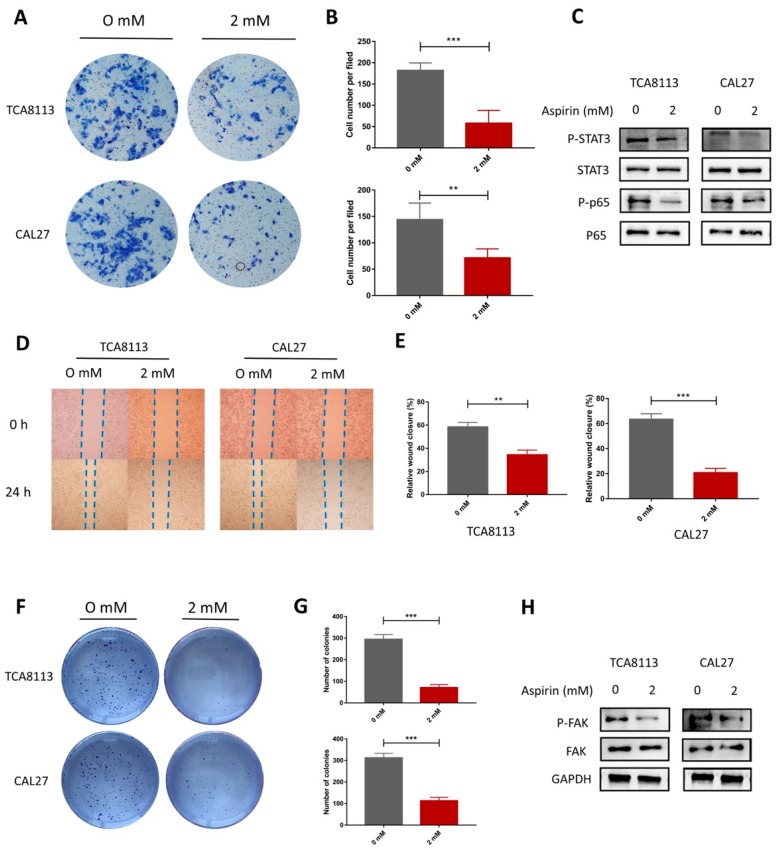
Aspirin decreased the migration and invasion in the TCA8113 and CAL27 cells. (**A**,**B**) The representative pictures of the Transwell invasion assay in the TCA8113 and CAL27 cells with ASA treatment. (**C**) The ASA treatment decreased the phosphorylation of Stat3 and p65. (**D**,**E**) The ASA treatment exhibited a slower scratch closure rate by the wound-healing detection in TCA8113 and CAL27 cells. (**F**,**G**) The colony formation was inhibited by ASA. The TCA8113 and CAL27 cells were incubated with aspirin for 14 days and the cell colonies were stained with crystal violet. The cell colonies with a diameter larger than 50 μm were counted using ImageJ software. (**H**) The phosphorylation of FAK were inhibited after they were ASA treated. Protein levels were detected by Western blotting using indicated antibodies, and GAPDH was used as a loading control. ** *p* < 0.01 and *** *p* < 0.005. The data were presented as the mean ± SD (*n* = 3).

To evaluate the impact of ASA on cell migration, wound healing assays and Transwell invasion assays were performed. It was found that ASA suppressed the migration of the TCA8113 and CAL27 cells (Figure 5D,E). Consistent with this finding, the Transwell invasion assays showed that ASA significantly weakened the invasion capacity of TCA8113 cells (Figure 5A,B). These results suggested that ASA played an important role in inhibiting the migration and invasion potential of the OSCC cells. According to the results of the bioinformatics, we detected the expression of FAK and p-FAK after the ASA treatment. As shown in Figure 5H (Figure S1D), the ASA significantly inhibited the activation of FAK, compared to the control group. To further investigate the mechanism responsible for the effects of ASA, we evaluated the effects of ASA on the expression and activation on the NF-κB and STAT3 signaling pathways in the TCA8113 and CAL27 cells. Compared to the control group, the ASA treatments significantly suppressed the phosphorylation of the P65 and STAT3 in TCA8113 cells (Figure 5C, Figure S1E,F). Similar effects were observed in CAL27 (Figure 5C). Meanwhile, the cell cloning was inhibited in the presence of ASA in the TCA8113 and CAL27 cells (Figure 5F,G).

## 3. Discussion

Aspirin and other NSAIDs were introduced as anti-inflammatory and analgesic drugs [20]. However, as the research progresses, other potential functions of aspirin are being uncovered, such as its role in cancer prevention [21,22]. Recently, a study that conducted a clinical trial reported that aspirin reduced the incidence and risk of metastasis in colon cancer patients [23,24]. Moreover, many studies reported that in cancers, including osteosarcomas, breast cancers, and myelomas, aspirin induces tumor cell apoptosis, reduces proliferation, and inhibits cancer cell migration and invasion in vitro [24,25,26]. In this study, we demonstrated that aspirin can inhibit the OSCC proliferation, increase apoptosis, and reduce migration and invasion using bioinformatics and in vitro studies.

We used bioinformatics to explore the changes after treatment with aspirin. From the DEGs between the control group and the aspirin treated group, we found that the genes involved in biological process of mitosis and the cell cycle were down regulated, which indicated that aspirin reduced cell proliferation of OSCC by blocking the cell cycle [27]. Furthermore, we found that 62 genes were highly-expressed in OSCC but were down-regulated by aspirin. Through a GO enrichment analysis, we found that these genes were involved in mitosis, the cell cycle, ECM catabolism, and cell adhesion, which play an important role in OSCC malignant phenotypes [28,29]. The PPI network also demonstrated the same results. The KEGG pathway analysis revealed that the extracellular matrix (ECM)-receptor, PI3K-Akt pathway, and focal adhesion molecules were significantly affected by aspirin. ECM proteolysis gives cancer cells an invasive capability, and is associated with migration in many types of cancers [30]. It has been reported that COL11A1 promotes tumor progression in EOC via ECM-receptor interactions [31]. The focal adhesion kinase (FAK) is an important mediator of proliferation, cell survival, and cell migration; the development of malignancy is often concerned with abnormal FAK activity [32]. It has been reported, regarding OSCC, that focal adhesion is related with cell proliferation [33]. The PI3K-Akt pathway plays an important role in cancer cell cycle progression, apoptosis, and neoplastic transformation [34], and has been involved in many of the mechanisms targeted by newer anti-tumor drugs [35]. These three pathways play an important role in OSCC tumorigenesis and all could be affected by aspirin. From our bioinformatic results, we propose that aspirin has anti-OSCC effects, which include inhibiting cell proliferation, promoting apoptosis, inhibiting cell migration, and invasion via the PI3K-Akt pathway and focal adhesion pathway.

Patients with metastatic or relapsing OSCC have poor prognoses; there is a strong need for new therapies. In the present study, we demonstrated that aspirin diminishes the growth and metastasis, and induces the apoptosis in the OSCC cell lines. Furthermore, we observed that aspirin exhibited an inhibitory effect on cell proliferation. Compared with the control group, the ASA treatment had significantly stronger effects on the inhibition of cell viability and colony formation in the TCA8113 and CAL27 cells. ASA also inhibited the phosphorylation of Akt, which is frequently activated in cell proliferation [36]. Since cell proliferation is closely related to the cell cycle, we further analyzed cell cycle distribution and found that aspirin significantly increased the cell population in the G0/G1 phase. Cell cycle regulatory proteins play a crucial role in the regulation of the cell cycle [37]. In tumor cells, the levels of p21 are usually low, which permits unchallenged proliferation. However, the levels rise following DNA damage, which inhibit cyclin D1 activity and induce cell-cycle arrest [38]. Our results showed that ASA could significantly suppress the expression of cyclin D1 and increase p21 expression. As the results show, the effects of aspirin on the CAL27 cells seemed weaker than those in the TCA8113 cells to some extent. We think this phenomenon can be attributed to the differences between the characteristics of different cell lines. We propose that the differences also exist among the other OSCC cell lines, and further experiments are needed to fully understand this phenomenon.

The FAK was significantly overexpressed and was notably hyper-activated in most of the solid tumors, including OSCC [39,40]. Our results showed that the phosphorylation of FAK was significantly suppressed by ASA in the TCA8113 and CAL27 cells. Cancer is characterized by the dysfunction of normal cell cycle regulatory mechanisms and the hyper-proliferation of cells [41]. The expression and function of the cell cycle regulatory proteins are commonly found to be altered in the OSCC, suggesting that studies of programmed cell death processes can lead to new strategies for the treatment of the tumor [42]. Furthermore, the suppression of apoptosis during carcinogenesis is also thought to play a pivotal role in the development and progression of some cancers [43]. Choi et al. reported that aspirin affected Bcl-2 translocation, and its phosphorylation in the nucleus triggers the apoptosis of breast cancer cells [25]. Our results also showed that the ASA treatment significantly increased the apoptotic rate of the cells in OSCC. The immunoblot experiments revealed that aspirin significantly decreased the expression of the anti-apoptotic protein Bcl-2 and increased the expression of pro-apoptotic proteins, Bax, and caspase3. Leemans et al. has proposed that the molecular landscape of HNSCC carcinogenesis is related to mutations associated mostly with tumour suppressor genes in cell cycle control, cellular growth and survival, WNT– β-catenin signaling, and epigenetics [44]. Moser et al. showed there are some key molecular players, such as the WEE1 kinase, that are critical for at least the G2–M checkpoint regulation and that are targetable by small-molecule inhibitors [45]. Thus, the exact effects and mechanisms of ASA on the expression of the tumour suppressor genes needs to be further studied.

It is considered a hallmark of cancer that cancer cells can spread by invading the adjacent tissue, often followed by local or distant metastasis [46]. The presence of cervical lymph node metastases is one of the most important prognostic factors for OSCC patients [47]. Therefore, we tested the effects of ASA on the cell migration and invasion in the OSCC cells. The results revealed that the ASA treatment significantly suppressed the ability of the migration and invasion. As for the effects on the cell cycle and apoptosis, the ASA increased the apoptotic cells and arrested the cells in G0/G1, leading to the inhibition of the cell migration and invasion. Moreover, various studies demonstrated that STAT3 enhances the cancer cells’ ability to invade and metastasize in response to integrating the signals from multiple extracellular stimuli [48,49,50]. We observed that ASA significantly inhibited the phosphorylation of STAT3 in the TCA8113 and CAL27 cells. In the last few decades, NF-κB signaling has attracted increasing attention in the field of cancer research. The global gene profiling analysis clearly indicated that NF-κB signaling is a major contributor to the metastatic progression of HNSCC and can be used as a prognostic biomarker of high-risk disease [16,51]. The immunoblot experiments showed that ASA significantly decreased the phosphorylation of p65 in OSCC cells.

## 4. Materials and Methods

### 4.1. Data Processing for Identification of DEGs

NCBI-GEO is a free database of microarray/gene profiles and next-generation sequencing (GEO, https://www.ncbi.nlm.nih.gov/geo/). Dataset GSE58162 were based on GPL10904 Platforms [Illumina HumanHT-12 V4.0 expression beadchip (gene symbol)]. GSE58162 included three groups of control OSCC cell line-SCC25 and three groups of aspirin treated SCC25 (2 mM aspirin). The GSE75538 was based on GPL18281 [Illumina HumanHT-12 WG-DASL V4.0 R2 expression beadchip]. It contained 14 OSCC samples and their matched normal samples. We used the GEO database online data analysis tool-GEO2R to analyze the two datasets to find out the DEGs. We set a cut-off criterion as *p* <0.05, and |logFC| >1 to find the DEGs. Next, we used a Venn diagram tool to identify overlapping DEGs in the two datasets.

### 4.2. Gene Ontology and Pathway Enrichment Analysis

The Database for Annotation, Visualization, and Integrated Discovery (DAVID, https://david.ncifcrf.gov/) was employed to perform the gene ontology enrichment analysis [52]. DAVID offers systematic and integrative functional annotation tools for investigators to discover the biological meaning behind the list of genes that are submitted. The gene ontology (GO) analysis includes the biological process (BP), cellular component (CC), and molecular function (MF). We submitted our lists into DAVID. A *p* <0.05 was regarded as of statistical significance, and the terms of the GO results were ranked by the *p*-value. A KEGG pathway enrichment analysis was carried out in the same way.

### 4.3. Protein–Protein Interaction (PPI) Network Construction

The function protein–protein interaction (PPI) analysis is important for the interpretation of the molecular mechanisms. The Search Tool for the Retrieval of Interacting Genes (STRING) [53] database (https://string-db.org/cgi/input.pl) was employed to construct the PPI network. The network was visualized in Cytoscape [54]. The APP, named the Molecular Complex Detection (MCODE) in Cytoscape, was utilized to screen the core module in the PPI networks. The APP Cytohubba was used to identify hub genes from the network.

### 4.4. Cells and Reagents

Human oral squamous carcinoma cell lines, TCA8113 and CAL27, were purchased from the Shanghai Cell Bank of Chinese Academy of Sciences (Shanghai, China). The two cell lines are both well-characterized and they both originated from tongue. The cells were cultured in Dulbecco’s Modified Eagle’s medium (DMEM), supplemented with 10% fetal bovine serum (Gibco, Grand Island, NY, USA) and 1% penicillin-streptomycin, at 37 °C in a humidified air containing 5% CO_2_. The antibodies to p-Akt, Akt, p-FAK, FAK, Bax, Bcl2, Caspase 3, p21, cyclin D1, p-p65, p-STAT3, and STAT3 were purchased from Abcam (Shanghai, China). The anti-GAPDH and p65 were purchased from Proteintech (Wuhan, China)

### 4.5. Cell Viability Assay

The TCA8113 and CAL27 cells were plated in 96-well culture plates at a density of 5 × 10^3^ cells per well. After 24 h incubation, the cells were treated with different concentrations of ASA for the indicated time periods. The cell viability was measured by the Cell Counting Kit-8 (CCK8, solarbio, Beijing, China) assays.

### 4.6. Invasion Assay

The Transwell invasion assay was used to detect the effect of ASA on the invasion ability of TCA8113 and CAL27. Briefly, in the transwell cell culture chambers, 8 μM pore-sized transwell inserts in 24-well plate were coated with matrigel on the upper surface. Then, 750 μL of a medium containing 20% FBS was added to the lower chambers. The cells were treated with or without ASA for 24 h, and were subsequently suspended with reduced serum DMEM and were seeded onto the upper chamber of the wells. After 24 h of incubation, the cells that invaded through the matrigel membrane were fixed in methanol and stained with 0.1% crystal violet for 30 min. We gently removed the cells on the inner layer with a cotton swab. The filters were washed with PBS and images were taken. The invading cells were counted and photographed under a light microscope (Olympus BX53, Tokyo, Japan) at ×200 magnification in five fields.

### 4.7. Scratch Assay

A scratch assay was performed to analyze the cell migration in vitro. The TCA8113 and CAL27 cells were seeded in 6-well plates; once confluent, a perpendicular scratch was generated on the surface of the plate using a pipette tip, followed by an extensive washing with a serum-free medium to remove cell debris. Then, we incubated the plates with DMEM medium containing 1% FBS and treated cells with the indicated densities of ASA. The photographic images were taken, using a light microscope (Olympus BX53, Tokyo, Japan), of each well at the indicated time after the drug treatment.

### 4.8. Clone Formation Assay

The TCA8113 and CAL27 cells plated in triplicate at 1000 cells per well in 6-well plates were treated with 0 mmoL/L and 2 mmoL/L ASA for 24 h, and then cultured for 14 days. The cell clones were washed with PBS, and then fixed in methanol and dyed with 0.1% crystal violet, and the colonies that contained more than 50 cells were counted.

### 4.9. Cell Cycle Analysis

The cells were plated in parallel on 35 mm^2^ culture plates, at a concentration of 1 × 106 cells/plate. After 24 h of serum starvation, the cells were exposed to ASA for 24 h and were harvested by trypsinization, washed in cool PBS twice, and placed in 75% ethanol overnight at 4 °C. After that, the cells were incubated in a solution with DNA-binding dye PI, RNase A (KeyGEN Biotech, Nanjing, China), for 30 min at 37 °C in the dark. Finally, red fluorescence from 488 mm laser-excited PI in every cell was analyzed by flow cytometer (Becton Dickinson, Franklin Lakes, NJ, USA). We used a peak fluorescence gate to discriminate the aggregates. The percentage of cells in G0/G1, S, and G2/M was determined from DNA content histograms using the BD Accuri C6 Plus (Becton Dickinson, Franklin Lakes, NJ, USA) software.

### 4.10. Apoptosis Flow-Cytometry Assay

The cell apoptosis was evaluated by flow cytometry using Annexin V-FITC/PI Kit (Hanbio, Shanghai, China). Briefly, the cells cultured in 12-well dishes were trypsinized and stained with PI-conjugated anti-Annexin V antibodies under darkness for 20 min at room temperature. Subsequently, they were analyzed by flow cytometry using Accuri C6 plus (Becton Dickinson, Franklin Lakes, NJ, USA).

### 4.11. Western Blot ANALYSIS

The cells were harvested after a corresponding treatment and were lysed in RIPA lysis buffer (Beyotime, Beijing, China) containing protease and phosphatase inhibitors. The protein concentrations were determined using BCA protein assays (Beyotime, Beijing, China). Of each sample, 30 μg was added and separated by SDS-PAGE, which was then transferred to a polyvinylidene fluoride membrane. The primary antibodies were added at 1:1000~5000 dilution and incubated at 4 °C overnight. Horseradish peroxidase-conjugated swine anti-rabbit IgG (DaKo, Glostrup, Denmark) was added and incubated at room temperature for 1 h. Western blot images were captured using a FluorChem E System (ProteinSimple, Santa Clara, CA, USA).

### 4.12. Statistical Analysis

All of the in vitro experiments were performed at least three times. All of the data are presented as mean ± SD. The differences between the two groups were performed using a t-test. The differences among the groups were tested by a one-way analysis of variance (ANOVA). Multiple-comparison tests were applied only when a significant difference was determined by the ANOVA. A *p* < 0.05 was deemed to be statistically significant.

## 5. Conclusions

In this study, we found that the ASA could downregulate 62 highly-expressed genes in OSCC, which regulate cell proliferation, cell adhesion, and ECM catabolism. We also provided new evidence proving that ASA promotes cell cycle arrest and apoptosis, and suppresses cell viability, invasion, and migration. However, further clinical and mechanical studies may be needed to validate the application of aspirin OSCC chemoprevention and treatment

## Supplementary Materials

Supplementary materials can be found at http://www.mdpi.com/1422-0067/19/7/2029/s1.

## Abbreviations

OSCC	oral squamous cell carcinoma
DEGs	differentially methylated genes
PPI	protein–protein interaction
GO	gene ontology
ASA	Aspirin
COX	cyclooxygenases
NSAID	non-steroidal anti-inflammatory drug
CC	cell component
BP	biological process
MF	molecular function
ECM	extracellular matrix
