# Supplementary material for: Aspirin is Involved in the Cell Cycle Arrest, Apoptosis, Cell Migration, and Invasion of Oral Squamous Cell Carcinoma
Source: Int J Mol Sci. 2018 Jul 12;19(7):2029. doi: 10.3390/ijms19072029 (PMC6073368; doi:10.3390/ijms19072029)
Supplement: Supplementary file 1 [file ijms-19-02029-s001.pdf]

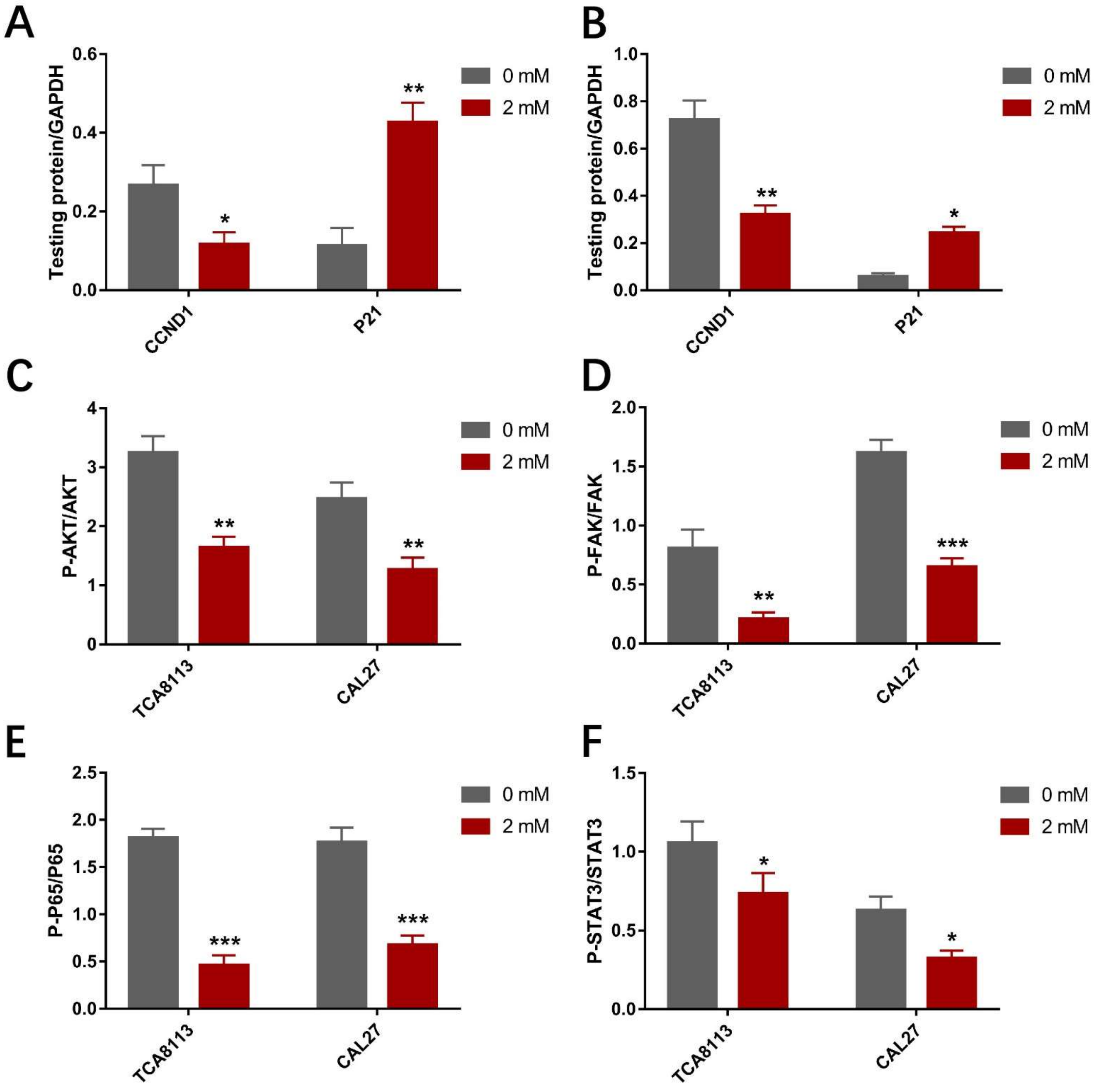

Figure S1 Qualification and statistics for the results of Western-Blot (A-B) The bar graph shows the gray value analysis of CyclinD1, and P21 for TCA8113 and CAL27 respectively. Data are mean ± SD (error bars) from three independent experiments. (C) The bar graph shows the gray value analysis of AKT and P-AKT. Data are mean ± SD (error bars) from three independent experiments. (D) The bar graph shows the gray value analysis of FAK and P-FAK. Data are mean ± SD (error bars) from three independent experiments. (E) The bar graph shows the gray value analysis of P65 and P-P65. Data are mean ± SD (error bars) from three independent experiments. (F) The bar graph shows the gray value analysis of STAT3 and P-STAT3. Data are mean ± SD (error bars) from three independent experiments. *P<0.05, **P<0.01, ***P<0.001

CDC20

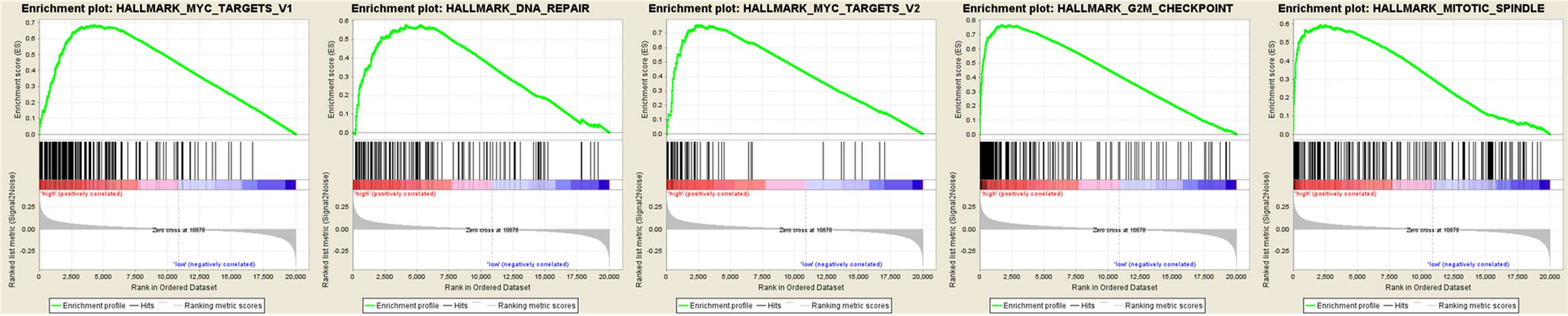
Enrichment plot: HALLMARK_MYC_TARGETS_V1
Enrichment plot: HALLMARK_DNA_REPAIR
Enrichment plot: HALLMARK_MYC_TARGETS_V2
Enrichment plot: HALLMARK_G2M_CHECKPOINT
Enrichment plot: HALLMARK_MITOTIC_SPINDLE
Enrichment score (ES)
Ranked list metric (Signal2Noise)
'high' (positively correlated)
Zero cross at 10878
'low' (negatively correlated)
Rank in Ordered Dataset
Enrichment profile
Hits
Ranking metric scores

CCNB2

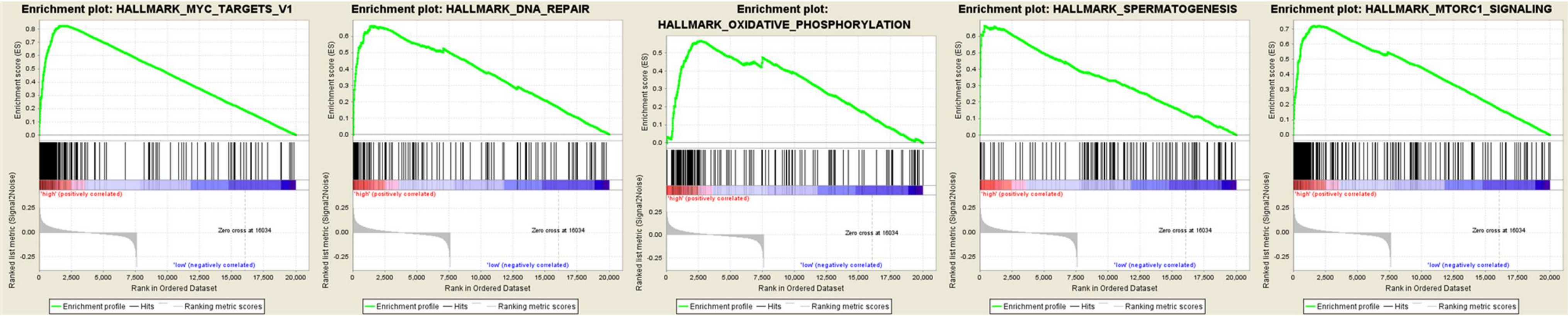
Enrichment plot: HALLMARK_MYC_TARGETS_V1
Enrichment plot: HALLMARK_DNA_REPAIR
Enrichment plot: HALLMARK_OXIDATIVE_PHOSPHORYLATION
Enrichment plot: HALLMARK_SPERMATOGENESIS
Enrichment plot: HALLMARK_MTORC1_SIGNALING
Enrichment score (ES)
Ranked list metric (Signal2Noise)
'high' (positively correlated)
Zero cross at 16034
'low' (negatively correlated)
Rank in Ordered Dataset
Enrichment profile
Hits
Ranking metric scores

BUB1

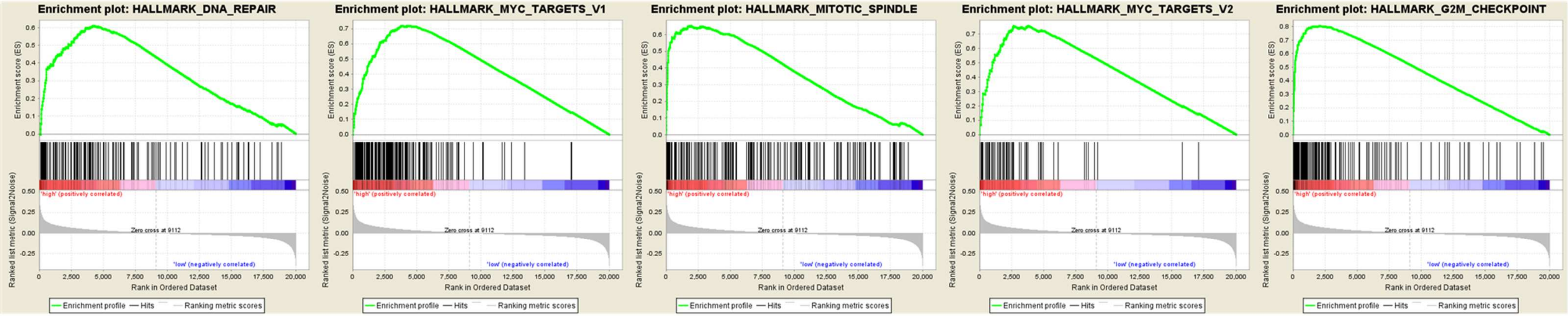
Enrichment plot: HALLMARK_DNA_REPAIR
Enrichment plot: HALLMARK_MYC_TARGETS_V1
Enrichment plot: HALLMARK_MITOTIC_SPINDLE
Enrichment plot: HALLMARK_MYC_TARGETS_V2
Enrichment plot: HALLMARK_G2M_CHECKPOINT
Enrichment score (ES)
Ranked list metric (Signal2Noise)
'high' (positively correlated)
Zero cross at 9112
'low' (negatively correlated)
Rank in Ordered Dataset
Enrichment profile
Hits
Ranking metric scores

KIF4A

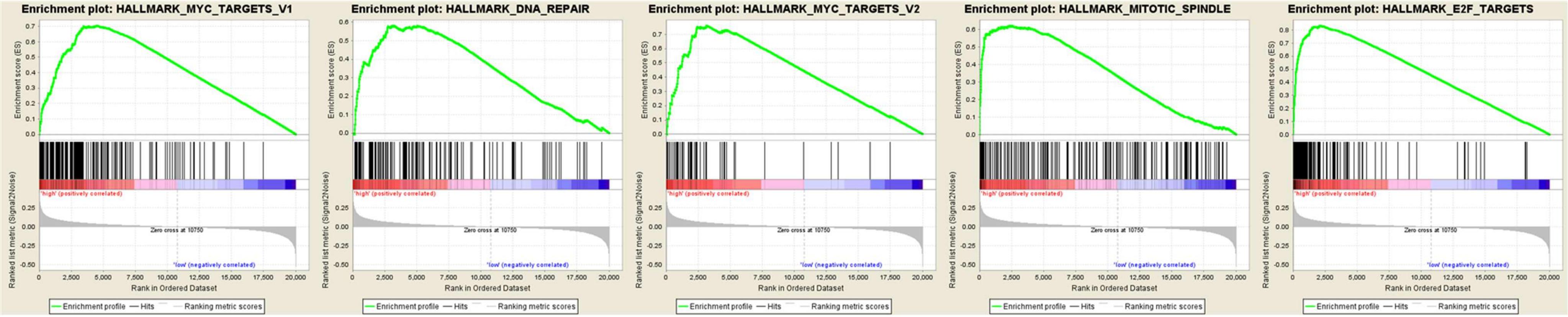
Enrichment plot: HALLMARK_MYC_TARGETS_V1
Enrichment plot: HALLMARK_DNA_REPAIR
Enrichment plot: HALLMARK_MYC_TARGETS_V2
Enrichment plot: HALLMARK_MITOTIC_SPINDLE
Enrichment plot: HALLMARK_E2F_TARGETS
Enrichment score (ES)
Ranked list metric (Signal2Noise)
'high' (positively correlated)
Zero cross at 10750
'low' (negatively correlated)
Rank in Ordered Dataset
Enrichment profile
Hits
Ranking metric scores

OAS1

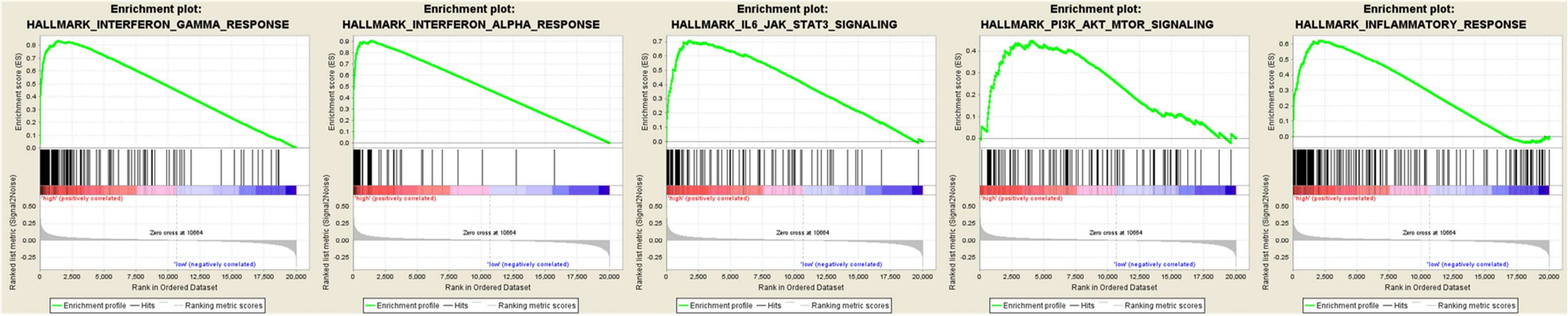
Enrichment plot: HALLMARK_INTERFERON_GAMMA_RESPONSE
Enrichment plot: HALLMARK_INTERFERON_ALPHA_RESPONSE
Enrichment plot: HALLMARK_IL6_JAK_STAT3_SIGNALING
Enrichment plot: HALLMARK_PI3K_AKT_MTOR_SIGNALING
Enrichment plot: HALLMARK_INFLAMMATORY_RESPONSE
Enrichment score (ES)
Ranked list metric (Signal2Noise)
'high' (positively correlated)
Zero cross at 10664
'low' (negatively correlated)
Rank in Ordered Dataset
Enrichment profile
Hits
Ranking metric scores

KIF14

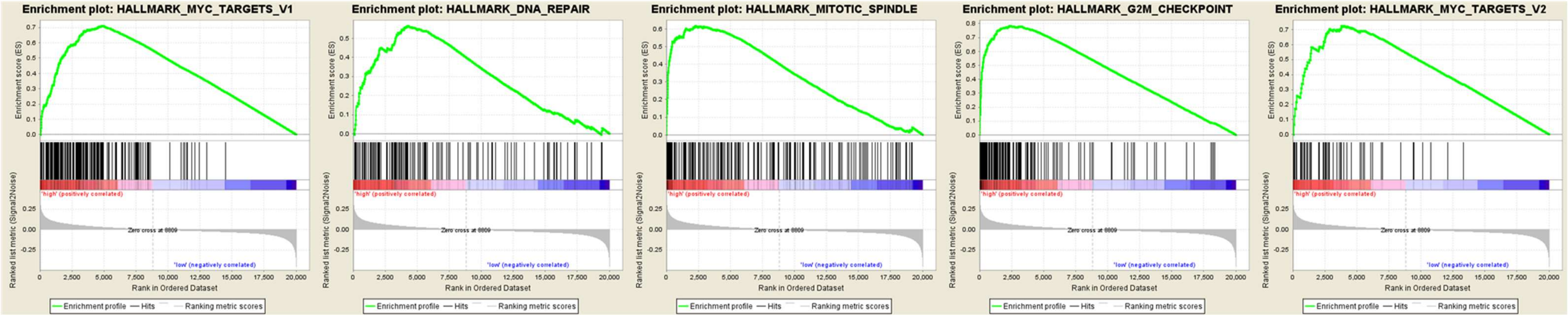
Enrichment plot: HALLMARK_MYC_TARGETS_V1
Enrichment plot: HALLMARK_DNA_REPAIR
Enrichment plot: HALLMARK_MITOTIC_SPINDLE
Enrichment plot: HALLMARK_G2M_CHECKPOINT
Enrichment plot: HALLMARK_MYC_TARGETS_V2
Enrichment score (ES)
Ranked list metric (Signal2Noise)
'high' (positively correlated)
Zero cross at 8809
'low' (negatively correlated)
Rank in Ordered Dataset
Enrichment profile
Hits
Ranking metric scores

AURKA

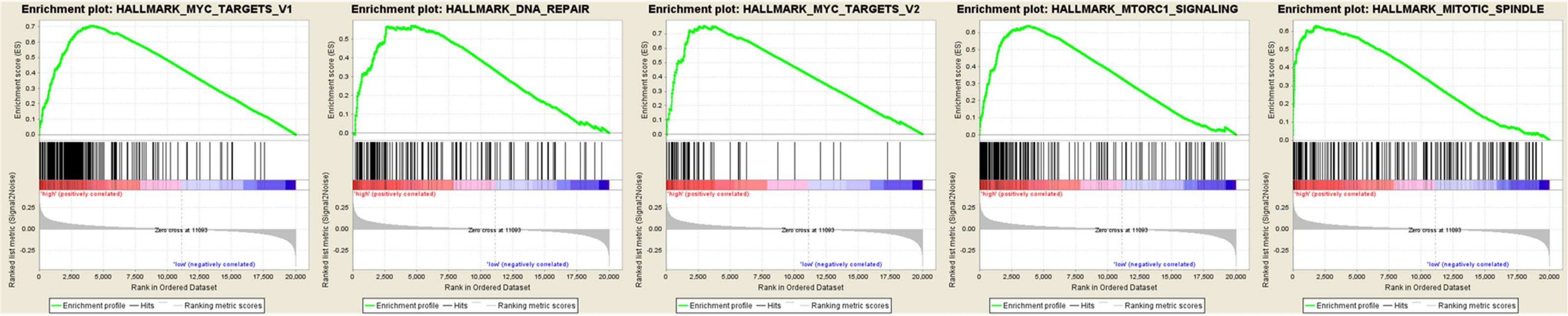

KIF20A

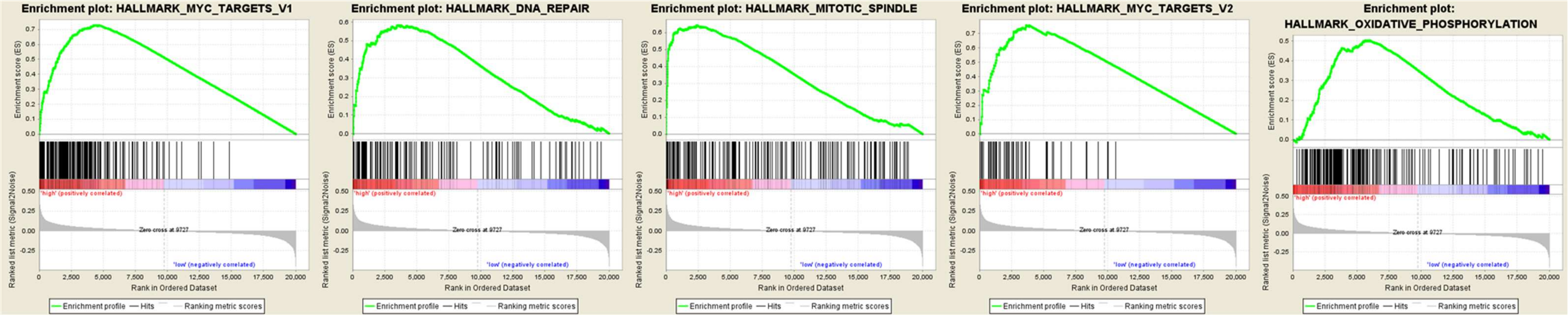

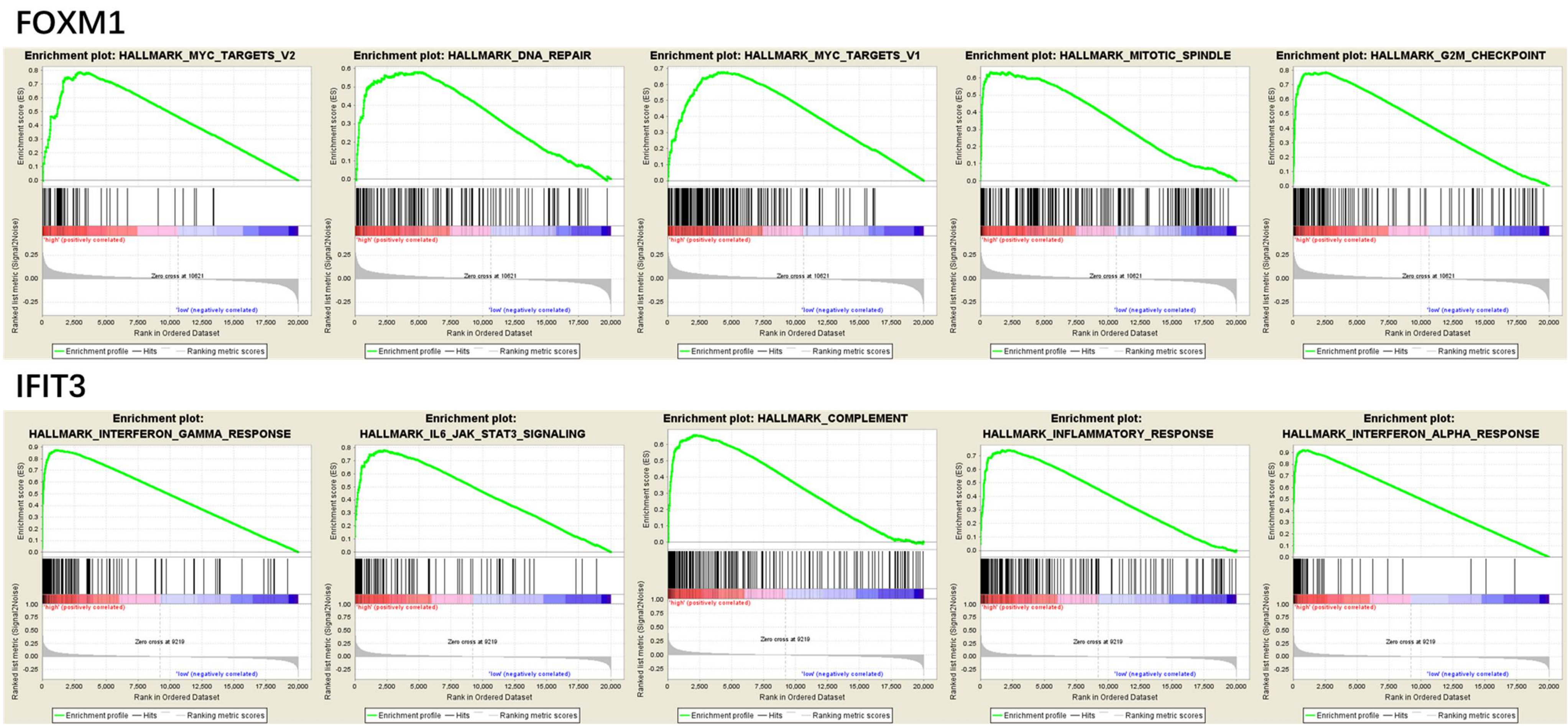

Figure S2 The GSEA analysis of the 10 hub genes to revealed their function. The dataset GSE30784 which contains 167 OSCC samples were downloaded from GEO database to perform GSEA analysis. The ten hub genes were divided into two groups according to their expression respectively (84 high expression group and 83 low expression group). The hallmark gene sets (h.all.v6.1.symbols.gmt) were used to show the function of the ten hub genes.
